# Amorphicity and Aerosolization of Soluplus-Based Inhalable Spray Dried Powders

**DOI:** 10.3390/pharmaceutics14122618

**Published:** 2022-11-27

**Authors:** Bishal Raj Adhikari, Shyamal C. Das

**Affiliations:** School of Pharmacy, University of Otago, Dunedin 9054, New Zealand

**Keywords:** dry powder inhaler, inhalation, powder, pulmonary drug delivery, soluplus, spray drying

## Abstract

Soluplus is a polymer that has been explored to prepare nanocomposites for pulmonary drug delivery and is non-toxic. However, its aerosolization attributes when spray-dried have not been investigated. Hence, this work aimed to investigate the aerosol performance of soluplus-based spray-dried powders. In addition, the potential use of leucine to improve the aerosolization of such particles was also investigated by including leucine at 10 or 20% *w*/*w*. 4% *w*/*w* salbutamol was used as a model drug in all the formulations primarily to aid quantification during aerosolization evaluation and for assessing the interaction between the drug and soluplus using infrared spectroscopy with the multivariate analysis approach of principal component analysis (PCA). Three formulations (4% salbutamol/96% soluplus, 4% salbutamol/86% soluplus/10% leucine, 4% salbutamol/76% soluplus/20% leucine) were prepared. The formulations were characterized in terms of solid-state, water content, particle size/morphology, and aerosolization. Similarly, two additional formulations (14% salbutamol/86% soluplus and 24% salbutamol/76% soluplus) were prepared to assess potential non-covalent interactions between salbutamol and soluplus. The formulations with only salbutamol and soluplus were amorphous, as evident from X-ray diffraction. Leucine was crystalline in the formulations. All the spray-dried formulations were irregular spheres with surface corrugation. The 96% soluplus powder showed an emitted fraction (EF) and fine particles fraction (FPF) of 91.9 and 49.8%, respectively. The inclusion of leucine at 10% did not increase the EF; however, an increase in FPF (69.7%) was achieved with 20% leucine. PCA of the infrared spectra suggested potential non-covalent interactions between salbutamol and soluplus. It hinted at the potential involvement of ketone groups of the excipient. This study concludes that soluplus-based spray-dried powder with or without leucine can potentially be utilized for pulmonary drug delivery. In addition, PCA can effectively be utilized in assessing interactions and overcoming limitations associated with visual assessment of the spectra of such formulations.

## 1. Introduction

Spray drying is an efficient particle engineering technique [1]. In recent decades, it has been extensively explored for the preparation of inhalable powders [2]. Such inhalable powders can broadly be categorized into two classes. The first class includes powders that are excipient-free, and the second class is those with excipients. Some of the common excipients which have been explored for pulmonary drug delivery in conjunction with particle engineering using a spray dryer include amino acids (e.g., leucine, valine, methionine, phenylalanine, glycine, histidine, lysine, and tryptophan), carbohydrates (e.g., lactose, maltose, trehalose, sucrose, and cyclodextrins), phospholipids (e.g., dipalmitoyl phosphatidylcholine), fatty acids (e.g., stearates) and polymers (e.g., polyvinyl alcohol, and chitosan). The use of excipients reflects a strategy to address different challenges in the development and optimization of inhalable powders. For example, amino acids have been used to improve aerosolization as well as chemical aerosolization of ceftazidime in the concentration of ~20% *w*/*w* [3]. Similarly, trehalose was used as a bulking agent in attempts to prepare inhalable dry powder of roflumilast, a potent anti-inflammatory agent [4].

**Figure 1:**
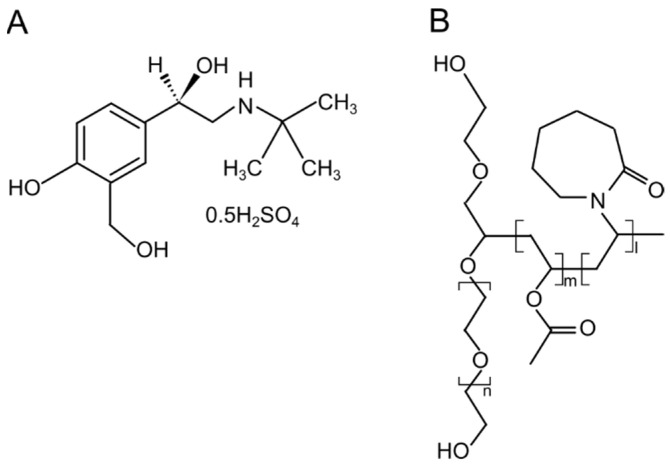
The molecular structures of (**A**) salbutamol sulfate and (**B**) soluplus.

Given that only a limited number of excipients are currently approved by the United States Food and Drug Administration (US FDA), there is increased interest in adopting excipients that have found usage in other drug delivery systems [2]. Soluplus (Figure 1) is a graft co-polymeric excipient built with polyethylene glycol, polyvinyl caprolactam, and polyvinyl acetate [5]. It has been used in the preparation of amorphous solid dispersions in attempts to improve dissolution behavior [6]. Due to its amphiphilic nature, this polymer is also known to increase aqueous solubility via micelle formation. Recently, this polymer is also being explored for pulmonary drug delivery. The preparation of lyophilized porous aggregates of insulin-loaded soluplus-based micelles has been reported [7]. In the study, although the in-vitro aerosol performance of the powder was not evaluated, the cytotoxicity of the formulation was assessed on bronchial epithelial (Calu-3), alveolar epithelial (A549), and macrophages (RAW 264.7) cell lines using 3-[4,5-dimethylthiazole-2-yl]-2,5-diphenyltetrazolium bromide) and lactate dehydrogenase assays. The formulation was reported to be non-toxic. In another study, rifampicin-loaded soluplus micelles were prepared, and the in-vitro nebulization capacity over 15 min was assessed using a jet nebulizer and next generation impactor (NGI) [8]. In the nebulization study, a total mass output of 72% was observed, and the low output was attributed to potential micelle agglomeration in the solution, which inhibited efficient aerosolization of the micelles. A fine particle fraction (FPF) of 57% was reported.

In the current context, although soluplus has been explored for pulmonary drug delivery via nanocomposite preparations, its potential as an excipient in engineering inhalable powders using a spray dryer, a proven inhalable particle engineering technique, remains unknown. Hence, the primary aim of this work was to scrutinize the aerosol performance and solid-state of soluplus-based spray-dried particles. In addition, the effect of including leucine, an established aerosolization enhancer, in the spray-dried powder formulation was also investigated. For this, salbutamol was used at a low concentration of 4% WW, primarily to aid in the quantification of powders during the aerosol performance assessment. In addition, it was used as a model drug to assess interactions in the drug-soluplus matrix using infrared spectroscopy in conjunction with a multivariate analysis approach of principal component analysis.

## 2. Materials and Methods

### 2.1. Materials

Salbutamol sulfate was obtained from Cambrex Profarmaco, Milan, Italy. Soluplus (polyvinyl caprolactam-polyvinyl acetate-polyethylene glycol graft copolymer) was supplied by BASF, Ludwigshafen, Germany. L-leucine and silicone oil were purchased from Sigma Aldrich (St. Louis, MI, USA). The organic solvents were from Merck, Darmstadt, Germany. Water for use was purified using continental water purifying system from Millipore Corporation, Burlington, MA, USA.

### 2.2. Preparation of Formulations Using Spray Dryer

The formulations (4% salbutamol/96% soluplus (4SS_96Solu_SD), 4% salbutamol/86% soluplus/10% leucine (4SS_86Solu_10Leu_SD), and 4% salbutamol/76% soluplus/20% leucine (4SS_76Solu_20Leu_SD)) were prepared using a mini spray dryer, Buchi B-290, from Buchi Labortechnik AG, Flawil, Switzerland (Table 1). Water was used as a solvent system for all the formulations, and a total feed concentration of 0.5% *w*/*v* was used in all cases. The operation was carried out with an atomization gas flow rate of 650 L/h, an aspiration rate of 40 m^3^/h, an inlet temperature of 155 °C, and a pump rate of 2 mL/min. A spraying nozzle with a diameter of 0.7 mm was used. In addition to the aforementioned formulations, spray-dried salbutamol, spray-dried soluplus, and other formulations containing salbutamol and soluplus in the ratios of 14:86 and 24:76 were also prepared for assessing interactions between the drug and excipient using infrared spectroscopy (Table 1).

### 2.3. Powder X-ray Diffraction

The solid-state of the formulations was evaluated using an X’Pert PRO MPD PW3040/60 X-ray diffractometer from Malvern Panalytical, Malvern, UK. A Cu K(alpha) radiation and rapid real-time multi-strip (RTMS) X’Celerator detector were used. A nickel plate was used as a beta filter. The powder was placed on an aluminum holder, and the measurements were performed with a scan from 5–35 degrees 2theta over 5 min. The diffractograms were collected using an X’Pert data collector and analyzed using the HighScore suite provided by the manufacturer.

### 2.4. Thermogravimetric Analyzer

The water content in the formulations was assessed using a thermogravimetric analyzer, Q550, from TA instruments, New Castle, DE, USA. Here, approximately 5 mg of the sample was spread on a platinum pan and heated at a rate of 10 °C/min up to 300 °C. The change in weight from ambient temperature up to 130 °C was considered as the water content in a formulation and analyzed using the TRIOS software supplied by the manufacturer. The experiments were performed under a constant flow of nitrogen (10 mL/min) to the heating furnace.

### 2.5. Modulated Differential Scanning Calorimetry

Approximately 5 mg of the sample was heated at a rate of 2 °C/min at a modulation amplitude of 0.16 °C and a modulation period of 30 s using Q500 differential scanning Calorimetry (TA Instruments, New Castle, DE, USA). A non-hermetic aluminum pan was used, and the experiment was performed under a nitrogen purge of 50 mL/min. The data were analyzed using the software (TRIOS) provided by the manufacturer. This technique was primarily used to investigate the glass transition temperature (Tg) of the salbutamol and soluplus.

### 2.6. Infrared Spectroscopy

Potential interaction between salbutamol and soluplus in the formulation matrix was assessed using an infrared spectrometer, Varian 3100, from Varian Inc., Palo Alto, CA, USA. Approximately 3 mg of the powder formulation was placed on the attenuated total reflection (ATR) accessory unit, and a total of 64 scans were collected (400–1900 cm^−1^) with a resolution of 4 cm^−1^. The spectra were collected, and the peak position was analyzed using the Resolution Pro software from Agilent Technologies Inc., Santa Clara, CA, USA.

### 2.7. Particle Size Analyser

The particle size of the spray-dried formulations was assessed using a laser diffraction analyzer, LA-950, from Horiba Ltd., Kyoto, Japan. The device allows volume-based particle size analysis with a fraction cell module. Here, approximately 0.5 mg of the sample was dispersed in 10 mL of ethyl acetate (pre-saturated with drug/excipients) via sonication. The data were analyzed using the LA-950 V2 software provided by the manufacturer. D50 represented the diameter below which 50% of the particles existed.

### 2.8. Scanning Electron Microscopy

The morphology of the spray-dried formulations was assessed using an electron microscope, Zeiss Sigma, from Carl Zeiss Inc., Oberkochen, Germany. Using a simple dusting method, the powders were spread on adhesive carbon tape. The particles were then coated with a very fine layer of gold/palladium alloy (80:20 *w*/*w* ) using a Quorum Q150TE turbo-pumped carbon coater (Quorum Technologies Ltd., Sussex, UK). The images were captured at an accelerating voltage of 5 kV.

### 2.9. In-Vitro Aerosolization

The aerosol performance of the prepared formulations was assessed using a cascade impactor, Next Generation Impactor (NGI), from Copley Scientific Limited, Nottingham, UK. The equipment was powered with a vacuum pump (HCP5), and the airflow control was achieved using a critical flow controller (TPL 2000), which was fined tuned to the desired flow rate using a flow meter supplied by Copley. A capsule-based Aerolizer was used as a model inhaler device to disperse the powder. Briefly, the aerosol performance of ~20 mg of a formulation was evaluated using the Aerolizer and NGI. Prior to actuation, all the stages were coated with a thin layer of silicone oil (viscosity = 10^−5^ m^2^/s (at 25 °C)) to mimic the thin fluid lining in the lungs. A flow rate of 60 L/min for 4 s was used in all cases. Following each test, the powder residues in the Aerolizer and various parts of NGI, including the mouthpiece, induction port, stages 1–7, and micro-orifice collector (MOC), were separately collected by rinsing with water and quantified using high-performance liquid chromatography (HPLC).

Quantification of salbutamol was performed using HPLC (LC-20AD) from Shimadzu, Kyoto, Japan. A C18 Synergi Fusion Column (250 mm × 4.6 mm, 5 μm, 80 Å, Phenomenex, CA, USA) was used with the machine. An injection volume of 20 µL was used, and the mobile phase consisted of 90% pH 3 buffer (ammonium acetate/acetic acid) and 10% methanol. The run time was 10 min, and the retention time was ~5 min. The absorbance was measured at 230 nm. The excipients, soluplus and leucine, did not interfere with the quantification of salbutamol. Standard solutions of 100, 50, 25, 12.5, 6.25, 3.12, 1.5, 0.75, and 0.4 µg/mL concentrations were used to prepare the standard curve (R^2^ ≥ 0.99) (Table S1). The limit of detection and the limit of quantification for the method were 0.3 and 1.1 µg/mL, respectively.

### 2.10. Statistical Analysis

The DescTools package was used to assess the statistical significance where ever required in Rstudio [9,10]. The analysis of variance (ANOVA) was used as the statistical tool, and Tukey’s honest significant difference (HSD) was used as the post hoc test to assess significance. A *p*-value of <0.05 was taken to be of significance.

IR spectra were evaluated using multivariate analysis and principal component analysis (PCA). The tool was used to scrutinize the potential non-covalent interactions between salbutamol and soluplus. PCA was performed with the ChemoSpec package (version 5.1.48) in Rstudio [10,11]. Before applying PCA to the spectral data, the data were pre-processed. Pre-processing involved baseline correction, peak normalization, and selection of the region of interest (400–1850 cm^−1^). The PCA was powered by singular value decomposition, and the bootstrap technique was used to perform the cross-validation.

## 3. Results and Discussions

### 3.1. Preparation of the Spray-Dried Formulations

Three formulations containing 4% salbutamol/96% soluplus (4SS_96Solu_SD), 4% salbutamol/86% soluplus/10% leucine (4SS_86Solu_10Leu), and 4% salbutamol/76% soluplus/20% leucine (4SS_76Solu_20Leu) were prepared (Table 1). The process yields of the different formulations varied from 18–34% (Table 2). The low yield was likely due to the poor efficacy of the cyclone separator in collecting the powder, as lighter/smaller particles would have a higher chance of being carried away with the airflow and escaping the separator. A significant powder deposition in the cloth filter beyond the cyclone separator was observed. The laboratory-grade spray dryers as the one used in this study, are known to exhibit poor yield due to the inefficacy of the cyclone separator in collecting powder particles [3,12]. A slight increase in yield with the use of leucine was observed and is consistent with results reported in the literature. For example, an increase in leucine concentration was associated with an increase in the process yield of the levofloxacin-leucine spray-dried particles [13]. The yield correlated well with the increase in particle size with the use of leucine, as in this study.

### 3.2. Characterization of the Formulations

Following the preparation of the various powders, a complete characterization was performed using XRD, particle morphology/size analysis, spectroscopic, and thermal techniques.

#### 3.2.1. Powder Amorphicity/Crystallinity

**Figure 2:**
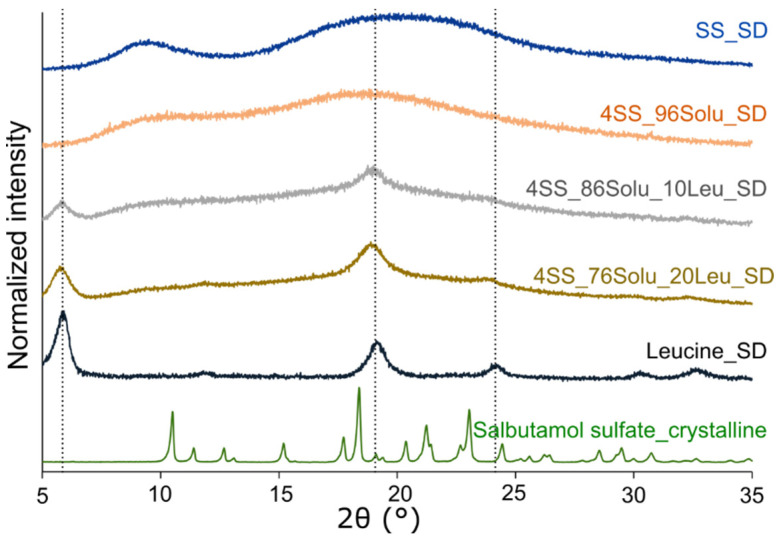
X-ray diffractograms of the various powders (salbutamol sulfate only (SS_SD), 4% salbutamol/96% soluplus (4SS_96Solu_SD), 4% salbutamol/86% soluplus/10% leucine (4SS_86Solu_10Leu_SD), and 4% salbutamol/76% soluplus/20% leucine (4SS_76Solu_20Leu_SD) and spray-dried leucine (Leucine_SD)).

X-ray diffractograms of the different formulations suggested that the formulation containing salbutamol/soluplus only (4SS_96Solu_SD) was amorphous; other formulations containing leucine (4SS_86Solu_10Leu_SD and 4SS_76Solu_20Leu_SD) were a mixture of amorphous and crystalline materials (Figure 2). In 4SS_86Solu_10Leu_SD and 4SS_76Solu_20Leu_SD, the peaks observed at 5.8, 18.8, and 23.9° 2theta corresponded well with peaks exhibited by spray-dried crystalline leucine suggesting that in the formulations, other components (salbutamol and soluplus) were amorphous, and introduction of leucine did not change the amorphicity of salbutamol or soluplus.

#### 3.2.2. Thermal Analysis

Thermogravimetric analysis of the powders showed that all the powders had a low water content of <1.9% *w*/*w* (Table 2). Such low water content for inhalable powders is desirable as moisture present in the formulation can lead to particle-sticking with or without transformation, which can adversely affect the aerosol performance of the powders [14]. 

Spray-dried amorphous salbutamol and amorphous soluplus showed Tgs of 120 °C and 78 °C, respectively (Figure S1). The observed Tgs of amorphous salbutamol and amorphous soluplus were similar with the values of 119 °C and ~70 °C, respectively, reported in the literature [15,16]. However, clear Tgs could not be observed for the formulations containing both salbutamol and soluplus.

#### 3.2.3. Assessment of Molecular Interactions Using Infrared Spectroscopy

**Figure 3:**
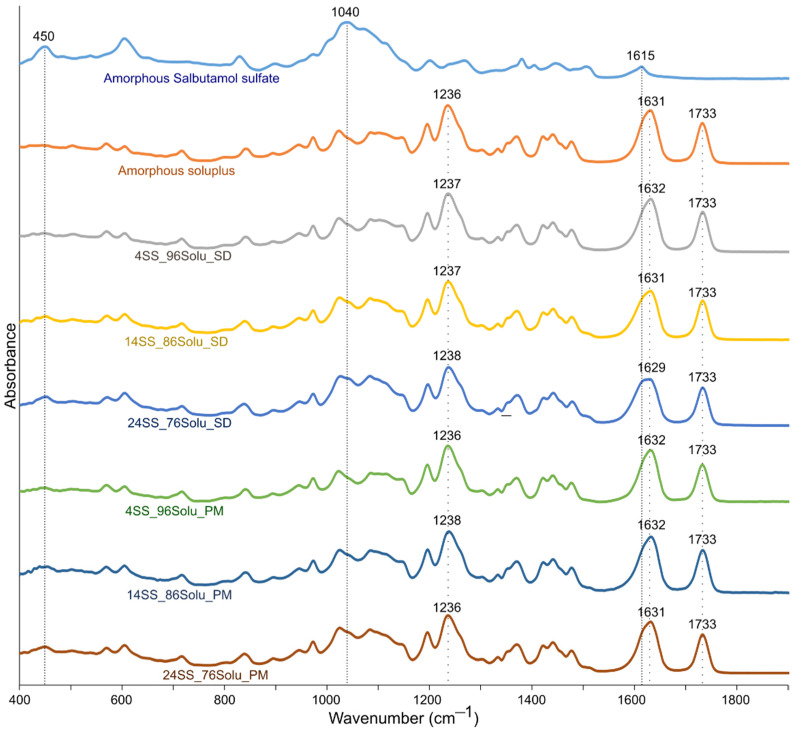
FTIR spectra of the various amorphous spray-dried powders (amorphous salbutamol sulfate, amorphous soluplus, 4% salbutamol/96% soluplus (4SS_96Solu_SD), 14% salbutamol/86% soluplus (14SS_86Solu_SD), 24% salbutamol/76% soluplus (24SS_76Solu_SD)) and their physical mixtures (4% salbutamol/96% soluplus (4SS_96Solu_PM), 14% salbutamol/86% soluplus (14SS_86Solu_PM), 24% salbutamol/76% soluplus (24SS_76Solu_PM)).

Potential molecular interactions between salbutamol and soluplus were assessed using FTIR. Here, salbutamol and soluplus were spray-dried in different ratios of 4:96, 14:86, and 24:76, and their spectra were compared with the spectra of their respective physical mixtures (Figure 3).

In the spectral region of 400–1900 cm^−1^, amorphous salbutamol sulfate showed multiple peaks (Figure 3). Some of them were 605, 829, 1040, 1201, 1267, 1506, and 1615 cm^−1^. Absorbance at 605 cm^−1^ likely originated from sulfate ion bending [17]. The peaks at ~1000 cm^−1^ likely originated from sulfate ion stretching, primary alcohol (C–O) stretching, and secondary alcohol (C–O) stretching. The peaks at ~1200 cm^−1^ and ~1250 cm^−1^ likely corresponded to phenol (C–O) stretching and primary/secondary alcohol (O–H) in-plane bending, respectively. The peak at 1615 cm^−1^ was likely due to secondary amine (N–H) bending. Similarly, amorphous soluplus showed characteristic peaks at 1023, 1085, 1196, 1236, 1631, and 1733 cm^−1^. Peaks ~1000–1250 cm^−1^ likely corresponded to a primary alcohol (C–O) stretching, ether C–O stretching, tertiary amine C–N stretching, and primary alcohol (O–H) in-plane bending. The peaks at 1631 and 1731 cm^−1^ potentially originated from the C=O groups in the molecule.

**Figure 4:**
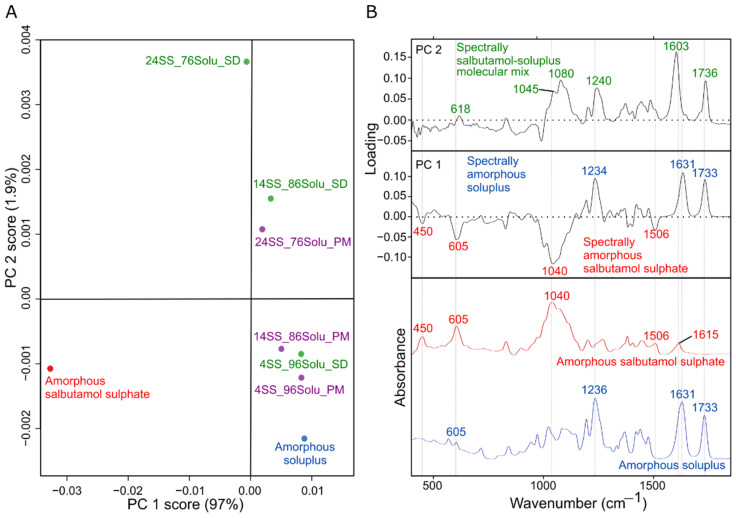
The principal component analysis of the FTIR spectral data of amorphous salbutamol sulfate, amorphous soluplus, and their spray-dried formulations (4SS_96Solu_SD, 14SS_86Solu_SD, and 24SS_76Solu_SD) and physical mixtures (4SS_96Solu_PM, 14SS_86Solu_PM, and 24SS_76Solu_PM) in the ratios of 4:96, 14:86, and 24:76. (**A**) Score plot of the spectral data. (**B**) Loading plots with FTIR spectra of amorphous salbutamol sulfate and amorphous soluplus as references.

The differences in spectra of the various spray-dried samples (salbutamol:soluplus—4:96, 14:86, and 24:76) and their respective physical mixtures were subtle. Therefore, the visual inspection did not suffice to evaluate potential non-covalent interactions (Figure 3). The mixture spectra were primarily dominated by features of amorphous soluplus. This was expected as soluplus formed the major bulk of the spray-dried powders, 76–96% *w*/*w* . Hence, a multivariate analysis approach, PCA, was employed to scrutinize interactions between salbutamol and soluplus in the amorphous matrix. The generated PCA model encompassed 98.9% of the variance in the spectral data (Figure 4). The first principal component (PC 1) corresponded to 97% of the variance in the spectral data. The PC 1 axis reflected the change in the chemical composition of the spray-dried powders. The PC 1 positive space was dominated by peaks at 1234, 1631, and 1733 cm^−1^, attributes of amorphous soluplus. The second principal component (PC 2) corresponded to a 1.9% variance in spectral data. The spray-dried formulations scored higher along the axis compared to their respective physical mixture suggesting the PC 2 reflected the spectral difference originating from the formulation matrix (molecular or physical mix). The positive PC 2 space corresponded to features unique to the molecular mix matrix of salbutamol and soluplus. For example, features at 1603 and 1736 potentially corresponded to changes in the molecular environment of keto groups (C=O) of soluplus in spray-dried formulation compared to amorphous soluplus.

#### 3.2.4. Particle Size and Morphology

**Figure 5:**
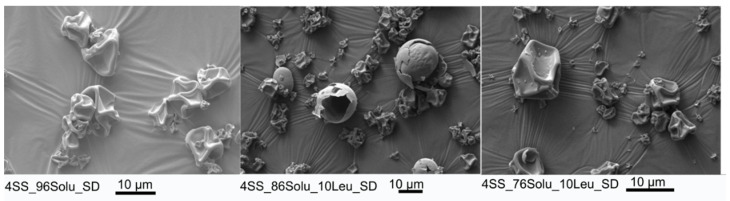
Representative SEM images of the spray-dried formulations (4% salbutamol/96% soluplus (4SS_96Solu_SD), 4% salbutamol/86% soluplus/10% leucine (4SS_86Solu_10Leu_SD), and 4% salbutamol/76% soluplus/20% leucine (4SS_76Solu_20Leu_SD)).

All formulations showed an average particle size of <5 µm (Table 2). D50 of the formulation containing 96% soluplus (4SS_96Solu) was 1.7 µm. D50 of the formulations with 10% (4SS_86Solu_10Leu) and 20% leucine (4SS_76Solu_20Leu) were 3.4 and 3.8 µm, respectively. An increase in particle size was observed with increasing concentration of leucine. This is likely related to the lower aqueous solubility of leucine compared to that of soluplus. Leucine likely achieves critical supersaturation concentration earlier during particle formation involving droplet drying. Critical supersaturation concentration refers to the concentration at which precipitation starts on the surface of the droplets; hence, it dictates the particle size [18]. The powder particles with high soluplus content (96%) (4SS_96Solu_SD) were irregular spheres in shape with large ridges/pits on the surface (Figure 5). The changes in morphology with the introduction of leucine in the formulations (4SS_86Solu_10Leu_SD and 4SS_76Solu_20Leu_SD) were subtle.

#### 3.2.5. Aerosol Performance of the Powders

**Figure 6:**
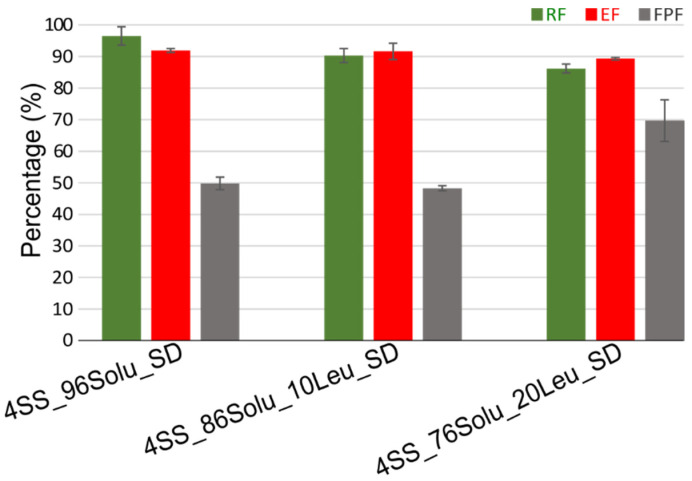
Aerosol performance of the various spray-dried formulations (4% salbutamol/96% soluplus (4SS_96Solu_SD), 4% salbutamol/86% soluplus/10% leucine (4SS_86Solu_10Leu_SD), and 4% salbutamol/76% soluplus/20% leucine (4SS_76Solu_20Leu_SD)).

The aerosol performance of the spray-dried formulations was assessed using NGI. At a flow rate of 60 L/min, the cut-off D50 values of stages 1–7 are 8.06, 4.46, 2.82, 1.66, 0.94, 0.55, and 0.34 µm, respectively. The recovered dose (RD) was the amount of drug which could be quantified from the Aerolizer and various stages parts/stages of NGI. It was expressed in percentage as recovery fraction (RF) (%) relative to the amount of powder loaded into the capsule. Emitted dose (ED) was the amount of drug which left the Aerolizer and was expressed in percentage as EF (%) relative to RD. Fine particle dose was the amount of drug present in particles with aerodynamic diameter ≤5 µm. It was calculated by interpolation of graph plotting cumulative mass in different stages of NGI and aforementioned cut-off D50 values of the respective stages. It was expressed relative to ED as FPF in percentage. Mass median aerodynamic diameter (MMAD) was also calculated using a similar approach. Hence, the evaluation of the aerosolization performance was based on the evaluation of RF, EF, and FPF (Figure 6, Figure S2, and Table S2). The RF was >85% in all cases. The formulation with high (96%) soluplus content, 4ss_96Solu, showed an EF of 91.9% and an FPF of 49.8%. The MMAD was 3.9 ± 0.2 µm. A high EF suggested that the powder is dispersible and easily fluidized. The corrugated surface, as evident from SEM images (Figure 5), contributed to the high EF. In general, surface asperities are desired for inhalable particles. Such asperities can improve the dispersibility of the powder bed by reducing the effective distance between two particles and decreasing the interparticulate interactions and agglomeration strength between particles [19]. In addition, individual particles, when aerosolized, experience higher drag which reduces the terminal velocity [20]. A lower terminal velocity improves the chances of avoiding upper respiratory tract deposition via inertial impact and increases its chances of reaching the lungs. This likely contributed to the observed FPF. The inclusion of leucine into the formulations (4SS_86Solu_10Leu and 4SS_76Solu_20Leu) did not foster an increment in EF; however, an increment in FPF to 69.7% was observed only in the case of the formulation containing 20% leucine (4SS_76Solu_20Leu). The MMAD of the formulation containing 10% leucine (MMAD = 3.9 ± 0.1 µm) was similar to that of the formulation without any leucine. However, the formulation with 20% leucine has a MMAD of 2.4 ± 0.4 µm. Amino acids such as leucine are known to improve the aerosolization of particles either by the introduction of surface asperities or by decreasing cohesive forces between particles as they are intrinsically less cohesive [2]. Here, as the change in surface asperity was subtle, the latter mechanism seems amenable. Nevertheless, it was intriguing to observe that no improvement in FPF was observed with the use of 10% leucine, suggesting a minimum concentration of leucine is required to observe the aerosolization enhancement capability of leucine when used with soluplus. While leucine is an established aerosolization enhancer, its efficacy is not universal. For example, when leucine was spray-dried with ivermectin at a concentration of 10% WW, it did not increase the FPF of ivermectin [21]. However, leucine improved the FPF of kanamycin from 48 to 73% when 5% leucine was used [22]. Such discrepancy in aerosolization enhancement behavior of amino acids is assumed to fragment from the multitude of factors such as particle size, density, surface asperity, surface enrichment, and relative intrinsic cohesive property of drugs/excipients used, which can dictate the aerosol performance of spray-dried particles [2,14,23,24].

## 4. Conclusions

Soluplus can be used to prepare inhalable microparticles in conjunction with the spray drying process. They tend to form corrugated particles, which are generally desired in inhalable powders as they can promote dispersibility as well as aerodynamic properties and facilitate deep lung delivery. In cases where attributes of soluplus as a solubilizer are desired with higher aerosolization performance, the use of leucine can be considered. Nevertheless, it is important to investigate the optimal amount of leucine required as a low leucine concentration of <10% WW may not suffice. If an assessment of non-covalent interactions between soluplus and drug is the aim, multivariate analysis such as principal component analysis should be considered as a visual inspection can be incomprehensive, as observed in this study.

## Figures and Tables

**Table 1:** The composition of the different spray-dried powders (4% salbutamol/96% soluplus (4SS_96Solu_SD), 4% salbutamol/86% soluplus/10% leucine (4SS_86Solu_10Leu_SD), and 4% salbutamol/76% soluplus/20% leucine (4SS_76Solu_20Leu_SD, spray-dried salbutamol, and spray-dried soluplus). 14SS_86Solu_SD and 24SS_76Solu_SD represent powders containing salbutamol and soluplus in the ratios of 14:86 and 24:76, respectively.

Powders	Salbutamol (% *w*/*w*)	Soluplus (% *w*/*w*)	Leucine (% *w*/*w*)
SS_96Solu_SD	4	96	-
SS_86Solu_10Leu_SD	4	86	10
SS_76Solu_20Leu_SD	4	76	20
Spray-dried salbutamol	100	-	-
Spray-dried soluplus	-	100	-
14SS_86Solu_SD	14	86	-
24SS_76Solu_SD	24	76	-

**Table 2:** The spraying drying process yield, water content, and particle size of the various formulations (4% salbutamol/96% soluplus (4SS_96Solu_SD), 4% salbutamol/86% soluplus/10% leucine (4SS_86Solu_10Leu_SD), and 4% salbutamol/76% soluplus/20% leucine (4SS_76Solu_10Leu_SD).

Formulation	Yield (%)	Particle Size (D50) (µm)	Water Content (% *w*/*w*)
4SS_96Solu_SD	18	1.7 ± 0.7	1.0 ± 0.3
4SS_86Solu_10Leu_SD	21	3.4 ± 0.3	1.5 ± 0.3
4SS_76Solu_20Leu_SD	34	3.8 ± 0.3	1.0 ± 0.2

## Data Availability

Not applicable.

## Supplementary Materials

The following supporting information can be downloaded at: https://www.mdpi.com/article/10.3390/pharmaceutics14122618/s1, Table S1: Linearity and accuracy of the chromatographic method used; Table S2: Aerosol performance of the various spray-dried formulations (4% salbutamol/96% soluplus (4SS_96Solu_SD), 4% salbutamol/86% soluplus/10% leucine (4SS_86Solu_10Leu_SD), and 4% salbutamol/76% soluplus/20% leucine (4SS_76Solu_20Leu_SD); Figure S1: DSC thermograms of (A) spray-dried salbutamol sulphate and (B) soluplus; Figure S2: Deposition behavior of different formulations 4% salbutamol/96% soluplus (4SS_96Solu_SD), 4% salbutamol/86% soluplus/10% leucine (4SS_86Solu_10Leu_SD), and 4% salbutamol/76% soluplus/20% leucine (4SS_76Solu_20Leu_SD) on various sections of next generation impactor (60 L/min) during in-vitro aerosolization assessment.
