# Supplementary material for: Amorphicity and Aerosolization of Soluplus-Based Inhalable Spray Dried Powders
Source: Pharmaceutics. 2022 Nov 27;14(12):2618. doi: 10.3390/pharmaceutics14122618 (PMC9787865; doi:10.3390/pharmaceutics14122618)
Supplement: Supplementary file 1 [file pharmaceutics-14-02618-s001.zip › pharmaceutics-2006240-supplementary.pdf]

pharmaceutics

MDPI

Supplementary Materials: Amorphicity and aerosolization of soluplus-based inhalable spray dried powders

Bishal Raj Adhikari and Shyamal C. Das

Tables

Table S1. Linearity and accuracy of the chromatographic method used.

**Linearity**				
Concentration (μg/mL)	Peak Area	Equation of regression	Correlation coefficient (R^2)	
100	20180330	y = 20171x - 8529.5<br>where,<br>y = peak area<br>x= concentration (μg/mL)	0.999	
50	10094430			
25	5051480			
12.5	2530005			
6.25	1269267			
3.12	637890			
1.50	311107			
0.75	157801			
0.40	89217			
**Accuracy**				
Sample	Area	Strength of sample (calculated)	Strength of sample (taken)	Recovery (%)
1	1718413	81.40	83.00	98.1
2	487956	23.08	23.50	98.2
3	19728	0.89	0.90	98.9
Mean				98.4
Standard deviation (%)				0.4
Relative Standard Deviation (RSD) or Coefficient of variation				0.4

Table S2. Aerosol performance of the various spray-dried formulations (4% salbutamol/96% soluplus (4SS_96Solu_SD), 4% salbutamol/86% soluplus/10% leucine (4SS_86Solu_10Leu_SD), and 4% salbutamol/76% soluplus/20% leucine (4SS_76Solu_20Leu_SD). RF, EF, and FPF represent recovery fraction, emitted fraction and fine particle fraction, respectively.

**Formulation**	**RF (%)**	**EF (%)**	**FPF (%)**
SS_96Solu_SD	96.5 ± 2.9	91.9 ± 0.6	49.8 ± 2.0
SS_86Solu_10Leu_SD	90.3 ± 2.2	91.6 ± 2.6	48.3 ± 0.8
SS_76Solu_20Leu_SD	86.2 ± 1.4	89.3 ± 0.4	69.7 ± 6.6

Figures

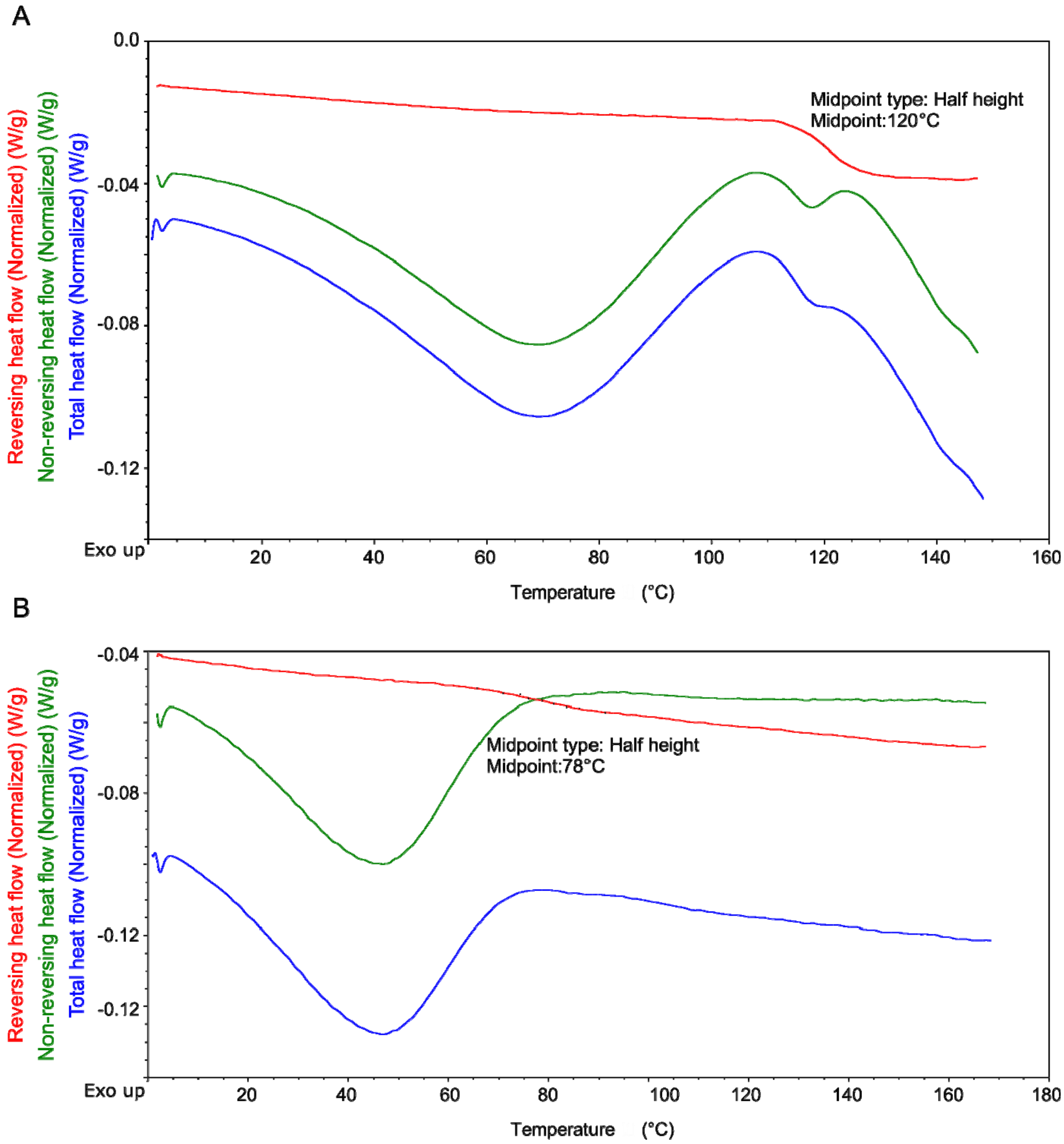

Figure S1. DSC thermograms of (A) spray-dried salbutamol sulphate and (B) soluplus.

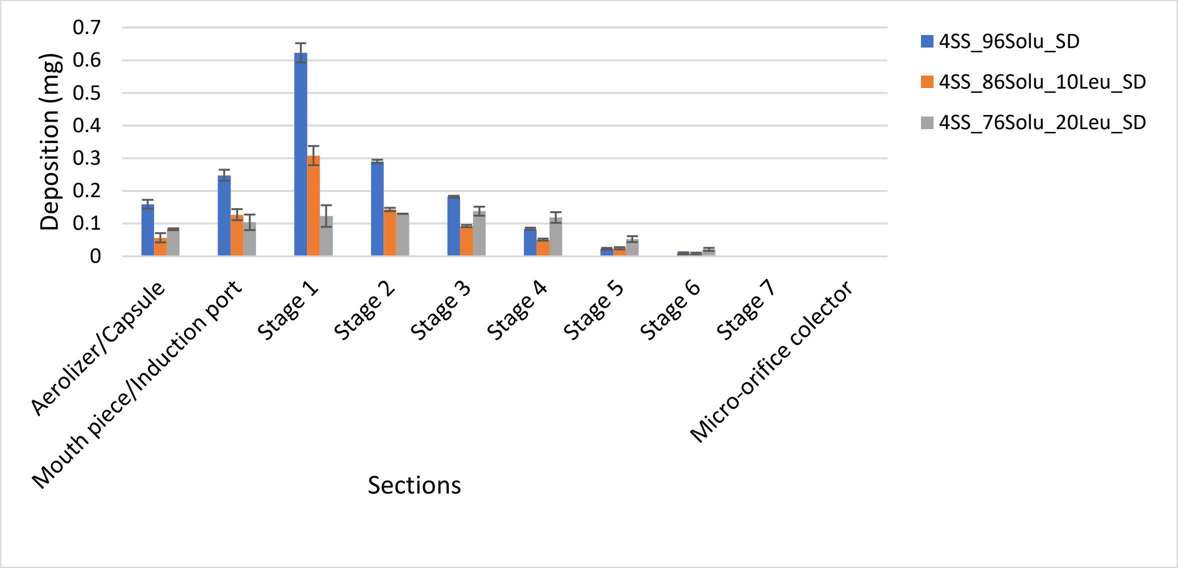

Figure S2. Deposition behavior of different formulations 4% salbutamol/96% soluplus (4SS_96Solu_SD), 4% salbutamol/86% soluplus/10% leucine (4SS_86Solu_10Leu_SD), and 4% salbutamol/76% soluplus/20% leucine (4SS_76Solu_20Leu_SD) on various sections of next generation impactor (60 L/min) during in-vitro aerosolization assessment.
